# Depression in Parkinson's Disease: The Contribution from Animal Studies

**DOI:** 10.1155/2017/9124160

**Published:** 2017-10-11

**Authors:** Jéssica Lopes Fontoura, Camila Baptista, Flávia de Brito Pedroso, José Augusto Pochapski, Edmar Miyoshi, Marcelo Machado Ferro

**Affiliations:** ^1^Department of Biology, Universidade Estadual de Ponta Grossa, Ponta Grossa, PR, Brazil; ^2^Department of Pharmaceutical Sciences, Universidade Estadual de Ponta Grossa, Ponta Grossa, PR, Brazil

## Abstract

Besides being better known for causing motor impairments, Parkinson's disease (PD) can also cause many nonmotor symptoms, like depression and anxiety, which can cause significant loss of life quality and may not respond to regular drugs treatment. In this review, we discuss the depression in PD, based on data from studies in humans and rodents. Depression frequency seems higher in PD patients than in general population, despite high variation in data due to diagnosis disparities. Development of depression in PD seems more likely to be caused by the nigrostriatal pathway degeneration than as a consequence of the awareness of disease prognostic, and it seems to be related to dopaminergic, noradrenergic, and serotoninergic synapses deficits. The dopaminergic role could be more significant, since it can modulate the release of the others, and its depletion is progressive, due to the degenerative feature of PD. Highly regarded in major depression, serotonin can be depleted in rats after nigrostriatal damage, but data from human patients are more conflicting. Animal studies can help in understanding the neurobiological mechanisms of depression in PD and the pursuit for more effective drugs for its treatment, but they lack the complexity of the disease progression, especially the nondopaminergic degeneration.

## 1. Introduction

Parkinson's disease (PD) is a progressively debilitating neurologic disorder that affects about 6 million people around the world [1]. The disease is mainly characterized by the progressive and irreversible degeneration of dopaminergic neurons localized at the substantia nigra pars compacta (SNc), on the mesencephalon, causing reduction in the striatal dopamine (DA) release [2–5].

The striatal DA deficit interferes directly in the basal nuclei's motor control circuitry, causing the most known PD symptoms: resting tremor, muscular rigidity, postural instability, and bradykinesia [3, 6, 7]. These symptoms and the consequent PD diagnosis occur when about 50% of the dopaminergic neurons at the SNc are already degenerated and striatal dopamine has been reduced in 80%. It must be considered that several other brain areas are also affected, some even before the mesencephalon. Among those structures, the mesolimbic pathway, the locus coeruleus, and the raphe nuclei can be damaged. This feature may be related to PD nonmotor symptoms [2].

Most PD patients can also present a series of nonmotor symptoms, sometimes even before the onset of the motor ones [8]. They significantly affect patient's life quality and many times do not respond to motor symptom treatments [9].

Nonmotor symptoms can include olfactive deficits, sleep disorders, autonomic disturbances, fatigue, pain, depression, and anxiety [1, 8, 10]. Additionally, patients may present cognitive dysfunction, which can evolve to dementia with compromised memory, thinking, and language [11]. PD patients suffer mostly from working memory and executive functions impairments, due to the dopaminergic nigrostriatal and mesocortical depletion, while the episodic memory and language are better preserved [12, 13]. This is confirmed by animal studies in which the nigrostriatal lesion affects cued and very short-term memory tests, while long-term spatial memory seems to be more dependent on the hippocampus integrity [14, 15].

Depression or anxiety symptoms are common in PD patients and are frequently associated [16]. Menza and coworkers reported depressive behavior in 92% of PD patients diagnosed with anxiety, as well as anxious behavior in 67% of PD depressed patients [17]. Those symptoms are implicated as the highest causes of poor life quality among PD patients, affecting their daily activities and increasing incapability more severely than the motor symptoms, even when in their advanced stage [18–20].

## 2. Depression in Parkinson's Disease

Depression is more frequent in PD patients than in general population [21, 22]. Incidence (4 to 75%) and prevalence (2.7 to 90%) of depression in PD patients in published studies vary substantially due to differences in depression definition or diagnostic criteria (i.e., a patient diagnosed with minor depressive symptoms or dysthymia by some authors and not included in the prevalence rates could be classified as depressed by others) [22]. Most of those studies do not correlate the depression prevalence with period of life of PD patients [22, 23]. In one study, patients with PD onset before 50 years of age presented higher frequency of depression than older-onset patients [23]. Despite its known high incidence and impact on life quality, there are no specific diagnostic criteria for depression in PD. Most diagnoses are based on the major depression criteria of the Diagnostic and Statistical Manual of Mental Disorders (DSM-V) [24, 25]. Besides, the difficulty in making a proper diagnosis due to symptom overlapping must be pointed out, since depression, sleep disturbances, and cognitive deficits are also seen in nondepressed PD patients [26].

Studies report an increase in depression prevalence in PD patients even before the onset of motor symptoms, indicating that the depression cannot be explained by a behavioral reaction to the PD diagnosis but more probably as a direct consequence of the degenerative process [27–29]. Furthermore, around 20% of the patients are already suffering from depression when diagnosed with PD [30]. In a 25-year-long study, it was concluded that the risk for the development of PD was higher among depressed patients, considering then depression as a risk factor to the development of PD [31]. Other depression risk factors in PD have been proposed, like severe cognitive impairment, female gender, and motor symptoms onset before the age of 40 years [32].

Depression in PD shows distinct characteristics from major depression not related to PD. Symptoms as irritability, sadness, dysphoria, pessimism, and suicidal ideation (to consider suicide without necessarily trying) are more frequent in PD depressed patients, while guilt, self-blame, feelings of failure, and suicide attempts are less usual [33]. In fact, it is reported that only a small percentage of PD patients are afflicted with major depression (2–7%); most of them present light depression or only a few depressive symptoms [34]. These symptoms receive less attention than the motor disturbances and often are not properly treated, increasing the risk for greater morbidity, disability, and lower quality of life [9, 35, 36].

## 3. Biochemical Theory of Depression

Much is discussed about the probable mechanism that leads to depression in PD and whether it is related to other psychiatric symptoms like apathy and anxiety. Among the more accepted theories, Schildkraut proposed that depression is linked to a deficit in monoaminergic neurotransmitters in specific brain regions [37]. This is based mostly on the mechanism of action of the first and second generations of antidepressants, which is the block of norepinephrine and/or serotonin presynaptic reuptake, enhancing their transmission [38–40].

Apart from their action on reuptake transporters, some antidepressants act on serotoninergic receptors, reinforcing the importance of this neurotransmitter in behavior. Serotonin acts by activating 5-HT receptors, a family with 14 identified subtypes. Besides the well-studied 5-HT_1A_, 5-HT_7_ have been indicated to be involved in depressive behavior, and 5-HT_2C_ antagonists can be used to treat both PD and depression. Since those receptors modulate dopamine release in distinct brain regions, this neurotransmitter also can be considered to be related to depressive behavior [41, 42].

Besides noradrenaline and serotonin, dopamine may also be involved in depression pathophysiology. Dopaminergic agonists, like pramipexole, efficiently improve depressive behavior in PD patients. It is suggested that this improvement is related to the D3 receptors stimulation, present on the mesolimbic system and participating in mood and behavior modulation [34]. The dopaminergic influence on depression is also suggested by the high therapeutic effectiveness of bupropion, a dopamine and noradrenalin reuptake inhibitor, useful regardless of whether it is prescribed alone or with other antidepressants [43].

A curious find is that high-frequency stimulation (HFS) of the subthalamic nucleus (STN), which is successfully used to treat movement disability in advanced Parkinson's disease, can cause or worsen depression and other psychiatric effects on patients [44].

## 4. Animal Models of PD

Since PD neurobiology is not fully understood, the use of animal models to improve the understanding of its etiology, pathophysiology, and molecular mechanisms is still of significant importance [45]. Also, these models became very useful to evaluate the efficacy of potential treatments in preclinical studies [46].

There are many forms of PD animal models, but, basically, they can be divided into two groups: models in which a neurotoxin (natural or synthetic) is used to kill dopaminergic neurons and the genetic models in which mutations in PD-related genes are induced [47]. Among the neurotoxins used, they can be either reversible, like reserpine, or irreversible, as 1-methyl-4-phenyl-1,2,3,6-tetrahydropyridine (MPTP), 6-hydroxydopamine (6-OHDA), paraquat, and rotenone. Irreversible toxins are usually preferred [45, 48–50].

Neurotoxin-based models provoke the degeneration of striatal pathway [51]. The infusion of different toxins on the same site may cause different responses. As an example, MPTP causes less dopaminergic cell death than 6-OHDA in rats [14, 52]. On the other hand, it is suggested that these toxins have different mechanisms of action, since MPTP causes a significant lesion only when infused at the SNc, a region rich in dopaminergic cell bodies, while 6-OHDA causes neuron degeneration when infused on cell bodies, axons (at the medial forebrain bundle), and even terminals (at the striatum) [50–53]. This reinforces the idea that 6-OHDA and MPTP can be taken by DA terminals, but 6-OHDA can damage DA neurons by other mechanisms, like extracellular oxidative stress generation [14, 52, 53].

6-OHDA lesions in the medial forebrain bundle, either unilateral or bilateral, promote the degeneration of the SNc and ventral tegmental area [54, 55]. On the other hand, lesions at the SNc are more selective to this region and therefore cause more modest dopaminergic depletion, affecting the nigrostriatal pathway without significantly damaging the mesolimbic pathway [56, 57]. A study using bilateral lesions suggested, based on forced swim test and elevated plus maze results, that SNc lesions caused more depressive and anxious behavior than ventral tegmental area lesions [58].

In addition, some works indicate that the bilateral lesion at the medial forebrain bundle can provoke severe motor impairment on rats, similar to the akinesia seen in more advanced stages of PD [59], while the SNc lesion is less severe and would mimic early stages of PD, when nonmotor symptoms are more evident [60]. Also, rats with severe motor deficit should not be used to study behavior, since their performance on swimming, drinking water, or walking through a maze will be compromised [61].

Genetic models often involve mice depleted from dopaminergic synapse-related genes. Although several genes have been related to the development of nonmotor signs of PD in humans (e.g., SNCA, LRRK2, VPS35, and Parkin), only a few studies have explored the influence of these mutations on depressive-like behavior in mice [62].

## 5. Behavioral Tests

To measure the behavioral alterations caused by the toxins or mutations, animal models are usually submitted to tests designed to evaluate depressive behavior in rats and mice [63].

In sucrose preference test, two bottles are offered to the animals: one containing water and the other containing 0,5 to 4% sucrose in water. Consumption of both is computed and two parameters are measured: total liquid consumption and preference for sweetened water over pure water. Reduction in the first is mostly related to motor disturbances or hypothalamic lesion, while the latter is related to anhedonia or loss of pleasure for formerly pleasant activities, which is a major depressive symptom and one of the few that can be evaluated in animals and related to what is reported by patients on questionnaires [63–65].

Another usual test for depressive behavior is the forced swimming test. Known as a behavioral despair test, it was developed by Porsolt et al. (1978) to evaluate antidepressant drugs [66]. Rats or mice are put in water in a cylinder with no chance to escape and tend to stop swimming after a while. A 15-minute-long pretest phase (stress generator) precedes the 5-minute-long test phase that happens 24 hours later. During the test, time spent on immobility over trying to escape is compared, and it is validated that antidepressant drugs reduce immobility and increase either swimming over water or climbing attempts on the cylinder walls. Based on that, it is considered that immobility mimics the giving up behavior seen in depressive patients [67, 68]. Also, it is proposed that serotoninergic antidepressant drugs are more prone to increase swimming, while noradrenergic drugs tend to promote climbing [69].

The tail suspension test is one of the most used tests to evaluate depressive behavior in mice. The test consists in suspending a mouse by its tail for 6 minutes. Similar to the forced swimming test, the animals tend to move and try to escape at first but later quit and stand immobile. Clinically effective antidepressant drugs promote an increase in the time spent trying to escape [70, 71].

## 6. Data from Animal Studies

A considerable amount of studies indicates that PD animal models present depressive behavior. Both unilateral and bilateral infusions of 6-OHDA on the SNc lead to a significant increase in immobility time on the forced swimming test and a decrease of sucrose consumption but not in total liquid consumption in rats when compared to control groups, characterizing a depressive-like behavior [72–75]. Santiago and coworkers (2014) [73] also reported a significant reduction in hippocampal serotonin, while Tadaiesky and coworkers (2008) showed a striatal serotonin reduction after a bilateral SNc 6-OHDA infusion, a toxin which is supposed to affect only catecholaminergic neurons [76]. Premotor symptoms of PD were also observed in a model generated by intrastriatal injection of 6-OHDA. The depressive-like behavior, observed in the sucrose preference test, was accompanied by a reduction in DA content in the dorsal striatum, indicating that dopaminergic deficit may be related to this behavior [77].

These data reinforce the cross-effect of the lesion on different neurotransmitters and the theory that depression in PD patients may be linked to alterations in serotoninergic systems and therefore is not strictly related to the dopaminergic degeneration. In addition, a study demonstrated reduced expression of tryptophan hydroxylase (TPH) and 5-HT_1A_ receptors in the dorsal raphe after rotenone injection, leading to a depressive-like behavior; this effect was improved with treadmill exercise [78].

In a study with noradrenergic drugs, pretreatment with desipramine minutes before the 6-OHDA infusion in mice did not prevent depressive behavior. On the other hand, reboxetine reduced lesion-induced depressive behavior on the forced swimming test [79].

A smaller variety of studies with lesion on the medial forebrain bundle are available. Carvalho and coworkers (2013) reported depressive behavior in rats by lesion on this site, indicated by reduced sucrose consumption similar to SNc lesion studies. However, they also stressed that both bupropion, a dopamine and noradrenaline reuptake inhibitor, and paroxetine, a serotonin reuptake inhibitor, were not able to reverse the depressive-like state, differently from other studies [80].

When tested in rats, STN HFS reduces both dorsal and medial raphe nuclei stimulation, which in turn reduces serotonin release [81]. On the other hand, Faggianni et al. showed that STN HFS was able to reduce immobility time in dopamine depleted rats but was less effective when applied in dopamine, noradrenaline, and serotonin depleted rats. These data support the idea that PD patients suffer a multineurotransmitter depletion, not restricted to dopamine [82].

Recently, cannabinoid receptors activation has been reported to have neuroprotective and antidyskinetic effects on animal models [83–85]. In humans, polymorphisms of CB1 have been related to signs of depression in PD patients [86]. However, to the authors' knowledge, no study has showed the effects of CB1 modulators on mood alterations seen in either PD rats or mice models.

In addition to studies that use lesions, studies using genetic models of Parkinson's disease have also indicated the involvement of neurotransmitter deficits in depressive behavior. Signs of apathy were observed in mice with deficiency of vesicular monoamine transporter 2 (VMAT2) by reducing sucrose preference, becoming a potential study model for investigation of the neurobiology of depression in PD [87].

A recent study used* CD157* KO mice, a PD genetic model displaying depression- and anxiety-like behaviors, to explore the antidepressant and anxiolytic effects of selegiline, an irreversible monoamine oxidase-B (MAO-B) inhibitor. The administration of selegiline reduced immobility time and increased climbing time in the FST in mice with depressive-like behavior. The mice with depressive-like behavior showed decreases in striatal and hippocampal serotonin. The levels of striatal serotonin returned after single administration of selegiline [88].

In mice, Pitx3 depletion has presented both depressive behavior and motor dysfunction [89]. PINK1 knockout mice have been shown to develop cannabinoid CB1 receptor dysfunction, which could play a role in the development of familiar PD [90].

## 7. Data from Human Studies

It is suggested that dopamine may have a role in the development of depression in PD patients, due to its association with the dopaminergic denervation in regions like the ventral striatum and prefrontal cortex [91]. These data are reinforced by the fact that dopaminergic agonists like rotigotine and pramipexole, used to treat PD, reduce depression symptoms [92].

Histologic studies in PD patients also reported a loss of nondopaminergic neurons in structures that do not compose the nigrostriatal pathway, including locus coeruleus and the dorsal vagal nucleus noradrenergic neurons or raphe serotoninergic neurons [93, 94]. Considering this, nonmotor symptoms of PD as depression and cognitive dysfunction could be related either to dopaminergic deficits in mesolimbic or mesocortical pathways or to other neurotransmitter areas in other regions [10].

Through positrons and single-photon emission computed tomography (PET and SPECT, resp.), loss in the integrity of dopaminergic, noradrenergic, serotoninergic, and cholinergic systems has been demonstrated in the brains of PD patients [95]. While striatal dopamine depletion in striatal pathway was considered responsible for the motor alterations, reduced binding to noradrenaline and dopamine transporters at locus coeruleus and several limbic system regions like thalamus, amygdala, and the ventral striatum of depressing patients was associated with depressive behavior [96].

Considering the serotonin role in PD, results in human studies are less certain. On one hand, some studies showed a correlation between degeneration of serotoninergic neurons and depression in PD patients [97–99]. On the other hand, a recent study did not suggest this correlation, based on the lack of neuropathological differences between depressed and nondepressed PD patients at the dorsal raphe nuclei, amygdala, and cortical regions, proposing that depression in PD is related more to dopamine and noradrenaline than to serotoninergic system dysfunction [100]. There is also contradiction among neurochemical studies, existing studies showing a reduction in serotoninergic metabolites like 5-hydroxyindoleacetic acid (5-HIAA) in the cerebrospinal fluid of depressed patients [101, 102], while others indicate no difference between depressed and nondepressed patients [34].

Other studies report an impairment in the cholinergic system. A reduction of acetylcholinesterase activity in the cortex of PD depressed patients has been shown [103], and PD depressed patients presented a reduction in acetylcholine receptor binding in the cingulate cortex and fronto-parieto-occipital cortex [104].

In addition to neurotransmitters, a recent study has indicated that neuropeptides may be involved in the pathophysiology of depression in Parkinson's disease. Neuropeptide Y (NPY) and calcitonin gene-related peptide (CGRP) are neuropeptides abundantly present in brain and may have their expression altered in several affective disorders. Svenningsson and coworkers examined the levels of NPY, CGRP, and 5-hydroxyindoleacetic acid (5-HIAA), the major serotonin metabolite, in cerebrospinal fluid (CSF) from PD patients, with or without comorbid depression, and compared them to the levels in patients with major depressive disorder. The levels of NPY and CGRP were higher in PD patients with depression compared to major depressive disorder patients. However, there was no difference in 5-HIAA levels between groups, indicating that depression in Parkinson's disease and the major depressive disorder can be generated by different processes [105].

## 8. Concluding Remarks

The development of depression in PD patients is linked to neurodegeneration and is not only a consequence of the realization of the disease prognostic. This is supported by the number of DP patients who present depressive behavior before the motor symptoms onset and by the depressive-like behavior shown by rats lesioned in the nigrostriatal pathway.

Depression in PD is at least partly dopaminergic, since lesions specific to these neurons cause this behavioral alteration and it is reversed or even protected by dopamine reuptake inhibitors.

Other neurotransmitters like acetylcholine, noradrenalin, and serotonin could also be implicated, just like the case in major depression. In rats, their release can be modulated by dopamine and influenced by this system depletion. However, in humans, the degeneration of other brain areas and consequent deficit in these neurotransmitters could happen at the same speed or even before the dopaminergic neurons.

Taken altogether, animal studies are very useful to study the neurobiological mechanisms of depression in PD, but with the fact that most of them fail to mimic the neurodegeneration observed in the PD patients, there are still many gaps to be filled by future investigations.
